# A spontaneous keypoints connection algorithm for leafy plants skeletonization and phenotypes extraction

**DOI:** 10.3389/fpls.2025.1641255

**Published:** 2025-10-24

**Authors:** Zhen Wang, Xiangnan He, Yuting Wang, Chenxue Yang, Beilei Fan, Qingbo Zhou, Xian Li

**Affiliations:** Agricultural Information Institute, Chinese Academy of Agricultural Sciences, Beijing, China

**Keywords:** leaves skeletonization, angle difference threshold, curvature minimization, keypoints connection, phenotype extraction

## Abstract

**Introduction:**

Leaf phenotypes are key indicators of plant growth status. Existing deep learning–based leaf skeletonization typically requires extensive manual labeling, long training, and predefined keypoints, which limits scalability. We developed a training-free and label-free approach that connects spontaneously detected keypoints to generate leaf skeletons for leafy plants.

**Methods:**

The method comprises random seed-point generation and adaptive keypoint connection. For plants with random leaf morphology, we determine a threshold for the angle difference among any three consecutive adjacent points and iteratively identify keypoints within circular search neighborhoods to trace leaf skeletons. For plants with regular leaf morphology, we fit the skeleton trajectory by minimizing curvature. We validated the approach on vertical and front-view images of orchids (covering random and regular morphological cases) and extracted five phenotypic parameters from the resulting skeletons. Generalization was further assessed on a maize image dataset.

**Results:**

On orchid images, the proposed approach achieved an average curvature fitting error of 0.12 and an average leaf recall of 92%. Five orchid phenotypic parameters were accurately derived from the skeletons. The method also showed effective skeletonization on maize, indicating cross-species applicability.

**Discussion:**

By eliminating manual labels and training, this approach reduces annotation effort and computational overhead while enabling precise geometric phenotype calculation from skeleton-based keypoints. Its effectiveness on both randomly distributed and regularly shaped leafy plants suggests suitability for high-throughput plant phenotyping workflows.

## 1 Introduction

Leaf physiological and morphological phenotypes are associated with plant growth status (Kolhar and Jagtap, 2023; Li et al., 2020), such as plant height (Liu et al., 2023; Zhang et al., 2024), leaf shape (Li et al., 2020), leaves consistency and color (Nyonje et al., 2021). Phenotypic information can be extracted manually or through computer-based automated methods. Manual phenotype measurement based on rulers is time-consuming and difficult to accurately capture complex phenotypes (Cembrowska-Lech et al., 2023). The consistency of leaf morphology can only be perceived subjectively. Computer-based phenotyping generally involves skeletonization and phenotype extraction. Skeletonization refers to simplifying each leaf region into a centerline polyline for geometric property calculations. In this study, skeletonization simplifies leaves into keypoints and skeleton, enabling efficient and precise geometric calculations of phenotypes without considering complex leaf details. Automated keypoint detection and skeletonization using intelligent algorithms can quantify leaf phenotypes and reflect the growth status, providing more efficient and intelligent decision support for precision agriculture (Jiang and Li, 2020). Deep learning has therefore become a mainstay in image-based phenotyping, in part because transfer learning, domain adaptation, and self-/few-shot strategies can support generalization across datasets and conditions while reducing annotation needs (Li et al., 2023; Ogidi et al., 2023; Sheikh et al., 2024).

Deep learning‐based methods for keypoints detection and skeletonization have been widely applied to crop phenotyping, providing valuable insights into plant morphology. For instance, point cloud based phenotyping approaches—using data acquired via LiDAR (Zhang et al., 2024), Visual Structure From Motion (VisualSFM) (Zhang et al., 2024), or Multi-View Stereo (MVS) (Murata and Noshita, 2024)—offer detailed 3D reconstructions, although they often require specialized equipment and involve higher technical and economic costs. For image data, methods such as YOLOv7-pose have been employed to extract keypoints from individual rice plants to facilitate stem-leaf angle measurements (Seng et al., 2024). In addition, lightweight variants like YOLOv7-SlimPose—enhanced with modules such as GSConv and GSIN and utilizing modified loss functions like MPDIoU—have been applied to detect multiple keypoints on maize leaves and stems, enabling the extraction of phenotypes including plant height, leaf-stem angle, leaf length, and ear position (Gao et al., 2024). Similarly, AngleNet has been used to extract keypoints on maize leaves (targeting the midrib, stem, and near the leaf neck) to quantify leaf angles (Xiang et al., 2023), and a stacked hourglass network (SHN) has been applied for locating keypoints on soybean leaves to automatically compute distances and angles between them (Zhu et al., 2020). While predefined keypoint sets can be effective, they may be less flexible for multi-leaf species with variable leaf counts or heavy occlusion. Recent structure-aware pose and dense-keypoint models partially mitigate this but challenges remain in complex canopies. Notably, several recent pose frameworks in crops can infer flexible landmark sets or even recover skeletal topology under occlusion. For example, PFLO reconstructs field maize poses with a YOLO-based head, and the bottom-up DEKR-SPrior leverages structural priors to detect variable keypoints in dense organs, mitigating the limitations of strictly pre-annotated keypoint templates (Pan et al., 2025).

While these approaches have significantly advanced plant phenotyping, many pipelines still supervise a fixed set of landmarks—particularly in multi-leaf canopies, although topology-flexible models partly alleviate this constraint. This design can sometimes limit flexibility when dealing with multi-leaf plants that exhibit considerable variability, such as differences in leaf count or challenges arising from occlusion. To address more complex plant structures, alternative strategies have been explored. For example, CenterNet has been applied to leaf counting in beet plants with an arbitrary number of leaves (Weyler et al., 2021), although overlapping leaves occasionally lead to false positives or missed detections. Similarly, SDNet, which employs an encoder-decoder architecture combined with a structural reconstruction algorithm (SRA), has been used for multi-instance detection, leaf counting, and phenotyping in maize and soybean (Lac et al., 2021), yet its ability to extract detailed keypoint information might be constrained under highly complex scenarios. More recently, the DEKR-SPrior model has been proposed to enhance keypoint detection by increasing the number of detected points and integrating prior structural knowledge through cosine similarity, thereby improving discrimination in dense leaf regions (He et al., 2024). Additionally, a Point-Line Net based on the Mask R-CNN framework has been developed to recognize maize field RGB images and determine both the number and growth trajectories of leaves and stalks, achieving promising performance (81.5% mAP50) (Liu et al., 2024). It should be noted that cross-species deployment of deep networks often benefits from fine-tuning or domain adaptation rather than always requiring full retraining. Recent work shows that synthetic-to-real adaptation, contrastive/self-supervised pretraining, and few-shot transfer can substantially reduce labeling demands and improve robustness across domains (Lagergren et al., 2023; Shi et al., 2022; Zhang et al., 2021).

Classical skeletonization techniques have also been applied to plant leaves. Medial-axis based skeletons are attractive for their geometric interpretability but are notoriously sensitive to small boundary perturbations (each local change on the silhouette can spawn spurious branches), requiring aggressive denoising and topology repairs under occlusion or gaps in the mask (Bucksch, 2014). Morphology-based thinning pipelines, widely available in plant phenotyping toolkits, routinely produce barbs/spurs whose prevalence strongly depends on mask quality and must be pruned with heuristic rules, which propagates instability to downstream trait calculations (PlantCV Morphology Tutorial). Active-contour (snake) models have been used to segment and track leaves in time-lapse data. However, they demand careful initialization and shape priors and may converge to local minima in scenes with weak edges or strong overlap, which limits their ability to deliver midrib-aligned skeletons and a variable number of keypoints needed for phenotyping (De Vylder et al., 2011; Scharr et al., 2016).

In this study, a training-free, spontaneous keypoint-connection algorithm is proposed to overcome the limitations of boundary-driven or annotation-dependent skeletonization. “Training-free” means that no model parameters are learned and no annotated data are required. “Spontaneous” refers to the fact that keypoints are not predefined. Instead, candidate interior points are sampled at runtime and connected according to curvature, angle-difference, and convexity rules to yield a single polyline per leaf without using templates or skeleton priors. Leaf regions are first isolated by color thresholding and morphological operations. Instead of predefined keypoints, randomly sampled interior points are linked through a set of connection rules. For irregular morphologies, an orientation-guided local search with an adaptive angle-difference threshold incrementally traces keypoints while halving the search space at every step, whereas for regular morphologies a convexity-constrained curvature-minimization scheme yields smooth, midrib-consistent polylines. This algorithm is positioned as complementary to learning-based pipelines—particularly useful in annotation-scarce settings, for rapid cross-species deployment, or when the keypoint graph is unknown or variable—rather than as a universal replacement. By dispensing with fixed keypoint counts, skeleton templates, and lengthy training, the method remains robust to edge defects, partial occlusions, and variable leaf numbers, thereby generalizing across species and enabling direct, geometry-accurate phenotype extraction for leafy plants with complex architectures.

## 2 Materials and methods

### 2.1 Image dataset acquisition

**Figure 1:**
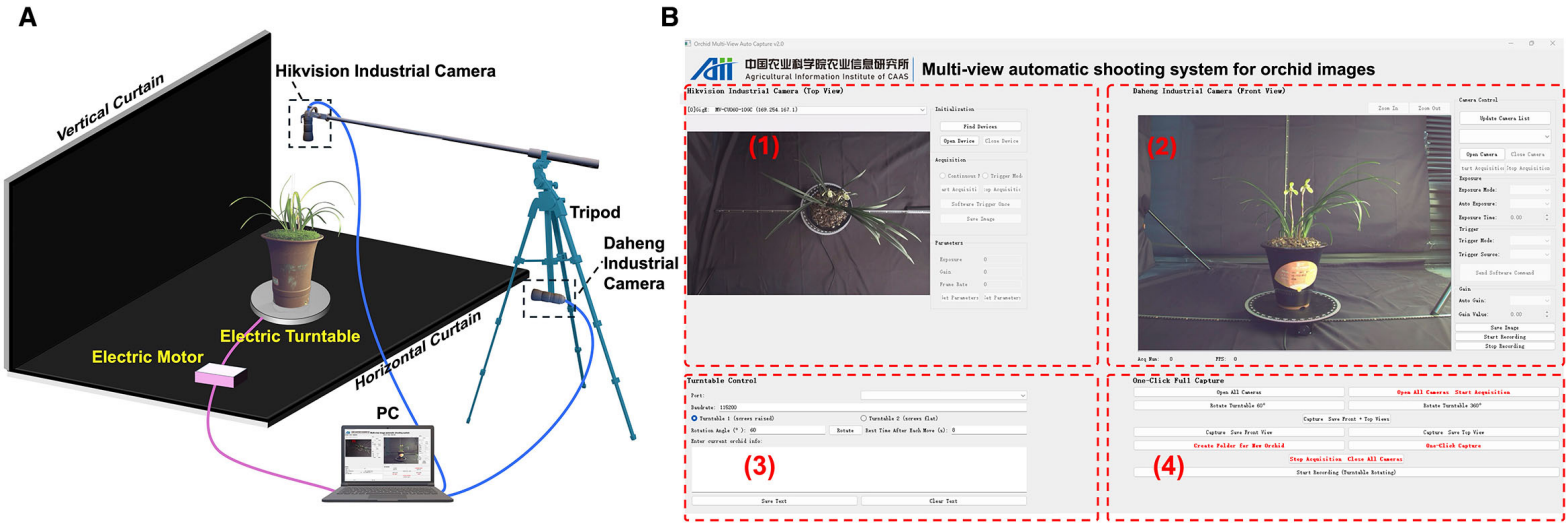
**(A)** Orchid image acquisition device. **(B)** Multi-view image automatic acquisition system interface with vertical view capture, front view capture, turntable control and auto-mated image acquisition.

The vertical (top view) and front views of leafy orchids effectively capture the randomness and regularity of leaf morphology, respectively (Guan et al., 2011; Rodrigues et al., 2013). To obtain these complementary perspectives, a multi-view automatic acquisition device was designed for capturing images of Cymbidium goeringii (*Rchb. f.*) (**Figure 1A**). While the front view provides more detailed phenotypic information and thus requires a higher resolution, the proposed algorithm is capable of processing images with varying resolutions.

Two industrial cameras were employed: a Daheng industrial camera (MER2-1220-32U3C, resolution: 4024×3036) for capturing front view images, and a Hikvision industrial camera (MV-CU060-10GC, resolution: 3072×2048) for capturing top view images. To verify the generalization ability of the proposed algorithm, a publicly available dataset of individual maize plants was used, which was captured using a Grasshopper 3 camera. The parameters of the three types of cameras are shown in **Table 1**. Notably, the distances between the cameras and the orchids were not fixed during data collection. Instead, these distances were dynamically adjusted based on the height and crown width of each orchid, ensuring that the entire plant was fully captured in both the top-view and front-view images.

**Table 1:** Key optical and sensor specifications of the imaging systems used for the orchid and maize datasets.

Camera (view)	Dataset	Native resolution	Pixel size (µm)	Lens & focal range
Daheng MER2-1220-32U3C (front)	Orchid	4024 × 3036 (12.2 MP)	1.85	16 mm C-mount fixed
Hikvision MV-CU060-10GC (top)	Orchid	3072 × 2048 (6 MP)	2.40	25 mm C-mount fixed
Grasshopper 3 GS3-U3-23S6C-C (12 side + 1 top)	Maize	2056 × 2454 (4 2 MP)	5.86	12.5–75 mm motorized zoom

The potted orchids were positioned at the center of a motorized turntable, which was controlled via serial communication with a multi-view image acquisition software (**Figure 1B**). The motorized turntable is controlled via RS-485 using the Modbus-RTU protocol through a USB-to-RS485 converter. Commands for absolute angle setting, step execution, and start–stop were issued from a Python 3.10 client using the pySerial library, with standard Modbus frames and a 9,600-baud 8-N-1 configuration. Module 1 captures the top view, Module 2 captures the front view, Module 3 controls the rotation angle of the motorized turntable for view selection, and Module 4 performs automated batch acquisition. This automated system enabled the turntable to adjust the viewing angle, capture images, and store data without manual intervention, thereby ensuring consistency and standardization throughout the data collection process. In total, 367 orchids with both vertical and front view images were collected during the Third China Spring Orchid Festival (Shaoxing, Zhejiang, February 22–25, 2024).

### 2.2 Image binarization and random point generation

**Figure 2:**
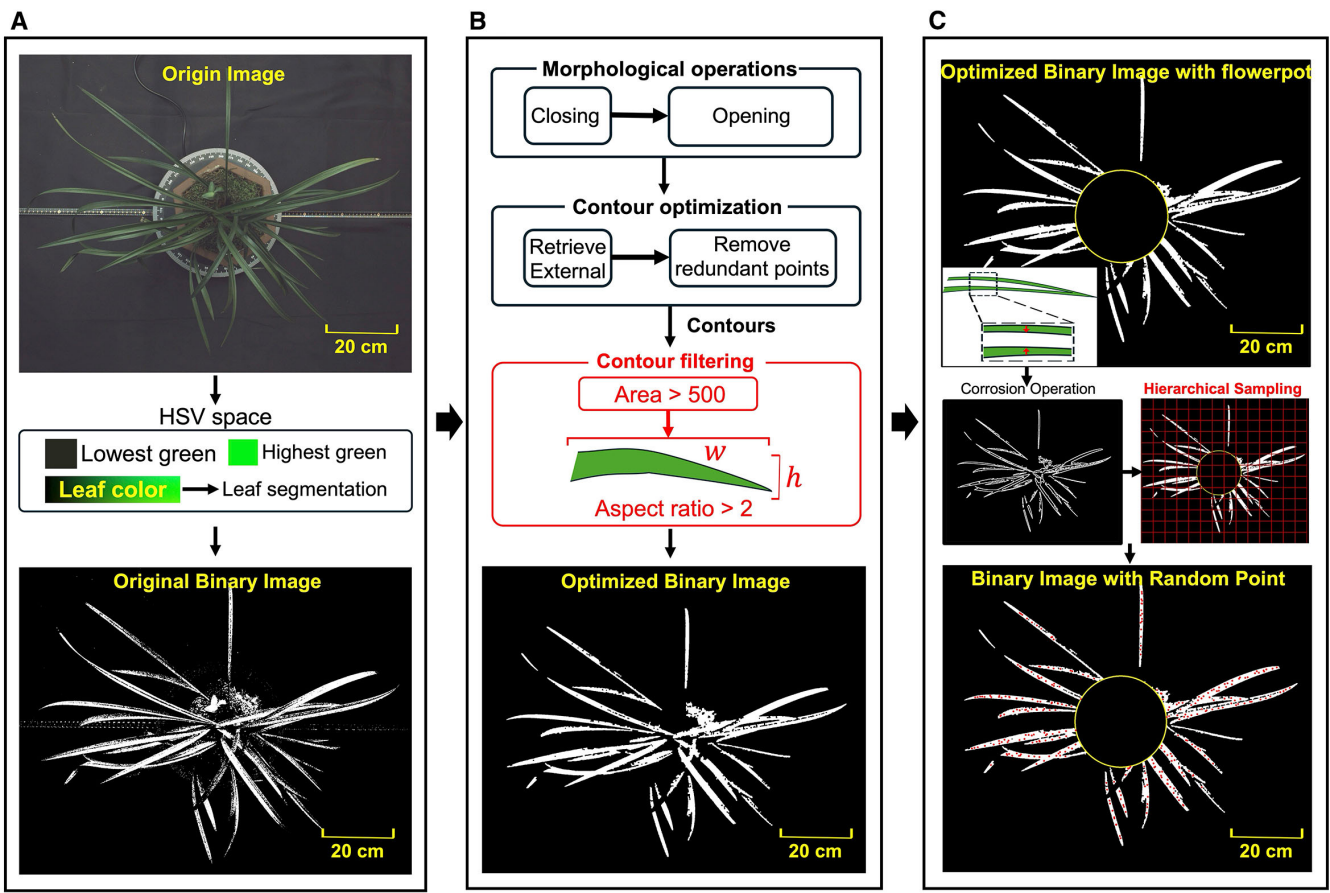
**(A)** Image binarization based on color threshold. **(B)** Binary images optimization based on morphological operations and contour features. **(C)** Random point generation based on corrosion operation and hierarchical sampling.

The hue channel in HSV (Hue, Saturation and Lightness) color space directly determines the color type (Hu et al., 2023; Shi et al., 2020), which facilitates accurate identification of regions of orchid leaves. The lower and upper thresholds for the green hues were established as the initial mask ( $Mask_{A}$ ), where the leaf regions were highlighted in white while the rest of the background was set to black (**Figure 2A**). However, due to the presence of background elements with colors similar to the leaves and the impact of lighting variation on leaves, the binarized image displayed noticeable gaps within the leaf regions and speckled noise in the background.

To eliminate the noise and accurately extract the contours of the leaves, a series of morphological operations were applied to the $Mask_{A}$ . Firstly, a morphological closing operation was used, followed by an opening operation, resulting in an improved mask $Mask_{A\prime }$ . The opening and closing operation were expressed as


Closing(A)=(A⊕B)⊖B

Opening(A)=(A⊖B)⊕B

where ⊖ and ⊕ represents erosion and dilation operation respectively. A is the binary image, B is a 5×5 ones matrix kernel that is used to probe and interact with A . The closing operation helped bridge small gaps and holes within the leaves (Le et al., 2020), while the opening operation effectively removed speckled noice within the background (Lei et al., 2019). Next, the binarized image is further refined based on the area and shape of the leaf contours. The contours of all connected regions within the white mask were traced pixel by pixel. Due to the presence of nested contours caused by noise, only the external contours were retained. To eliminate small contours unlikely to represent leaves, contours with an area smaller than 500 pixels were filtered out. The minimum bounding rectangle was then extracted for each contour, with its aspect ratio (the length ratio of longer side to shorter side) effectively distinguishing the elongated leaves from other objects. As the length of leaves are significantly longer than the width, contours with an aspect ratio greater than 2 were preserved. These contours, forming the set $S_{cont}$ , formulating a separate mask $Mask_{B}$ . The intersection of $Mask_{A\prime }$ and $Mask_{B}$ produced the final leaf mask $Mask_{leaf}$ , which highlighted the leaves in white against a black background, as shown in **Figure 2B**.

The keypoints on the orchid leaves were derived from randomly sampled points, with even distribution across the extracted leaf areas. Taking the vertical view image as an example, to simplify the computation, a circle $C_{pot}$ was drawn with the centroid of $Mask_{leaf}$ as the center and half the length of the shortest contour in the set $S_{cont}$ as the radius. This circle, $C_{pot}$ , generally covered the central part of the flowerpot in vertical view and served as a white mask representing the pot. As illustrated in **Figure 2C**, to ensure an even distribution of random points across the white regions of the leaf image, hierarchical sampling was employed to divide the segmented leaf area into 40×40 patches. A random point is sampled from each subregion, excluding those within $C_{pot}$ . Subsequently, morphological erosion algorithm is applied to reduce the boundary regions of the leaves (Yin et al., 2023), preventing points near the edges of the white areas from being selected as keypoints and ensuring that the generated random points were located close to the central skeleton of the leaves.

### 2.3 Initial keypoints recognition and search direction determination

**Figure 3:**
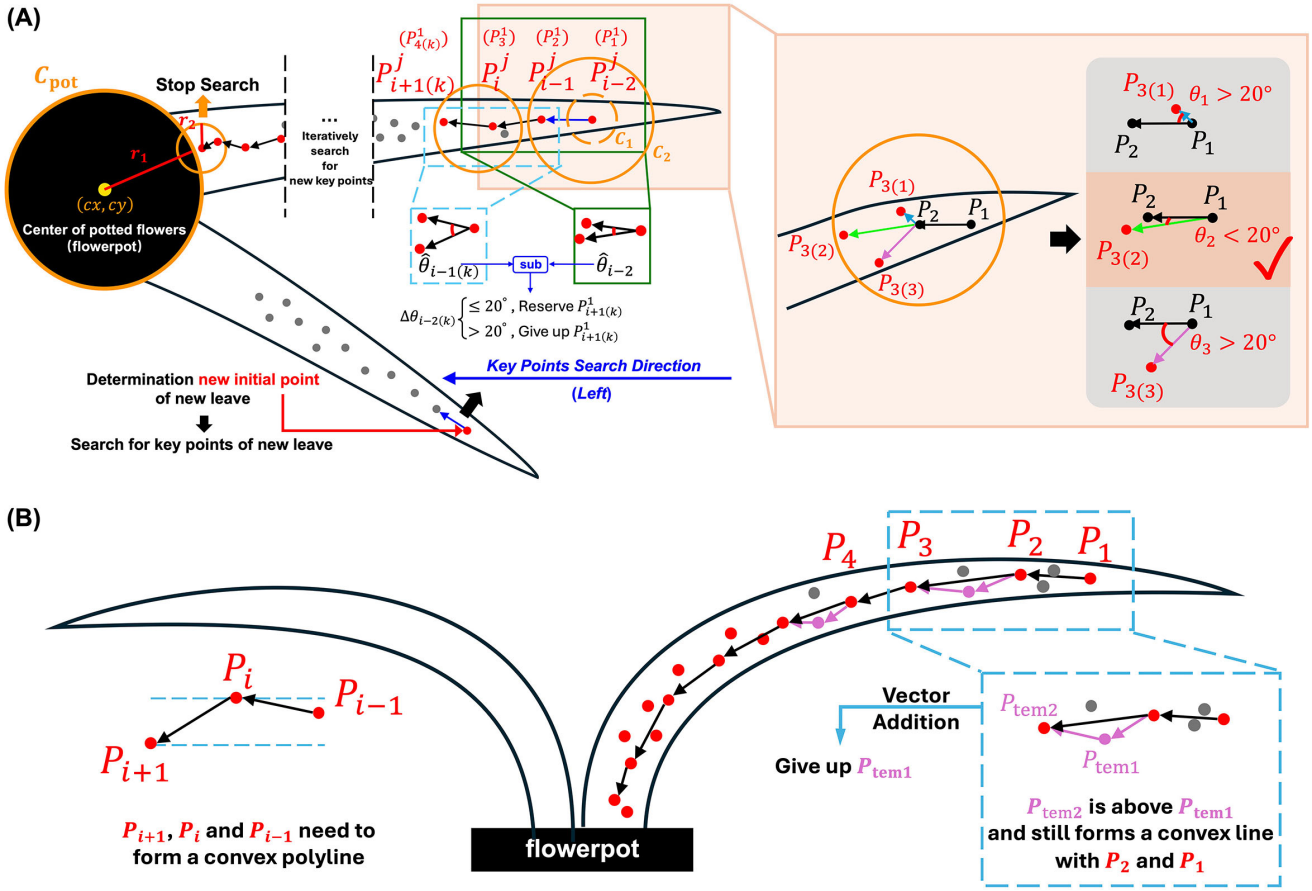
Keypoints determination method for vertical **(A)** vertical view and **(B)** front view.

For both random and regular morphological leaves, the outermost keypoints were firstly identified, then the search direction for subsequent keypoints was determined to reduce spatial complexity. The traversal of keypoints for the next leaf begins only after all keypoints of the current leaf have been identified. For each leaf, $P_{i}^{j}$ represents the i -th keypoints of the j -th leaf. $P_{1}^{1}$ was designated as the first keypoints of the first leaf (marked as visited), which has the maximum Euclidean distance from point $C_{pot}$ (closest to leaf tip), as illustrated in **Figure 3A**. $P_{2}^{1}$ was identified as the closest unvisited point to $P_{1}^{1}$ . To locate $P_{2}^{1}$ , a circular search area is iteratively expanded around $P_{1}^{1}$ until one or more unvisited points were found. If a single unvisited point is identified within the search area, it is designated as $P_{2}^{1}$ . If multiple unvisited points were found, the point closest to $P_{1}^{1}$ is selected as $P_{2}^{1}$ , which is then marked as visited, forming the vector $\vec{P_{1}^{1}P_{2}^{1}}$ .

The search for $P_{3}^{1}$ is also based on extending the circular search area centered on $P_{2}^{1}$ . However, given that the orientation of the leaf skeleton is determined, half of the random points within the circular search area are not candidate points for $P_{3}^{1}$ . The direction of the skeleton informs the search direction for subsequent keypoints, thereby reducing the search space for the next keypoint. The skeletal direction is determined by the relative positions of points $P_{1}^{1}$ and $P_{2}^{1}$ , specifically by comparing the absolute differences between the horizontal coordinates ( $P_{1x}^{1}$ and $P_{2x}^{1}$ ) and the vertical coordinates ( $P_{1y}^{1}$ and $P_{2y}^{1}$ ). This will result in more efficient search. The rules for determining the search direction for keypoints were shown in **Table 2**.

**Table 2:** The positional relationship between and determined the search direction for subsequent keypoints.

$\left|P_{1x}-P_{2x}\right|>\left|P_{1y}-P_{2y}\right|$		$\left|P_{1x}-P_{2x}\right|<\left|P_{1y}-P_{2y}\right|$	
$P_{1x}>P_{2x}$	$P_{1x}<P_{2x}$	$P_{1x}>P_{2x}$	$P_{1x}>P_{2x}$
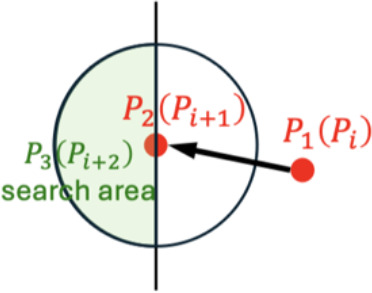	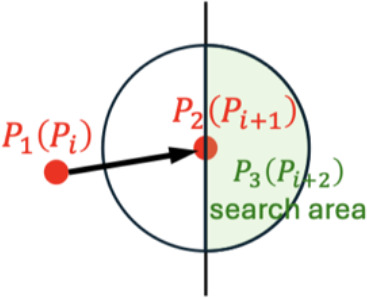	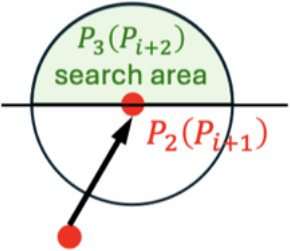	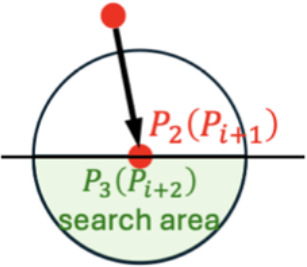

### 2.4 Remaining keypoints recognition for leaves with random and regular morphology

In vertical view of orchids, the uncertain growth trajectory of each leaf results in a random morphology. Therefore, based on the predetermined keypoint search direction, the optimal keypoints closest to the central skeleton must be adaptively identified. The determination of $P_{3}^{1}$ is based on the angle formed between vectors $\vec{P_{1}^{1}P_{2}^{1}}$ and $\vec{P_{2}^{1}P_{3}^{1}}$ , formulated as


$$S1_{\theta }=\left\{\theta_{P_{1}^{1}P_{2}^{1}P_{3\left(i\right)}^{1}}|\theta_{P_{1}^{1}P_{2}^{1}P_{3\left(i\right)}^{1}}=\text{arccos}\left(\frac{\vec{P_{1}^{1}P_{2}^{1}}\cdot \vec{P_{2}^{1}P_{3\left(i\right)}^{1}}}{\left|\vec{P_{1}^{1}P_{2}^{1}}\right|\cdot \left|\vec{P_{2}^{1}P_{3\left(i\right)}^{1}}\right|}\right)\times \frac{180}{\pi },i=1,2,3\right\}$$

$$\text{min}S1_{\theta }\leq 20^{\circ }$$

$$i=argminS1_{\theta },P_{3}^{1}\leftarrow P_{3\left(i\right)}^{1}$$

where $P_{3\left(i\right)}^{1}$ is the i -th candidate point of $P_{3}^{1}$ in circular search area. $\theta_{P_{1}^{1}P_{2}^{1}P_{3\left(i\right)}^{1}}$ is the angle between $P_{1}^{1}P_{2}^{1}$ and $P_{2}^{1}P_{3}^{1}$ . Within the left semicircle of the circular search area, there are three candidate keypoints, resulting in three angles. In order to effectively capture the natural curvature characteristics of orchid leaves along their main skeleton, we set a fixed threshold for the angular difference of candidate keypoints in our algorithm. Through statistical analysis and experimental validation on multiple orchid samples, we found that the local curvature variations of most leaves are confined within a narrow range. When the angular difference between a candidate keypoint and the current skeleton direction is less than 20°, the true turning points can be effectively identified while avoiding interference from noise and local anomalies. If the minimum angle in $S1_{\theta }$ is less than 20°, the corresponding candidate point is selected as $P_{3}^{1}$ . Otherwise, the circular search area is further expanded. This 20° threshold was chosen based on the observed morphological properties of orchid leaves and extensive empirical testing, which demonstrated that it provides a robust balance between sensitivity (capturing genuine turning points) and specificity (avoiding spurious points due to noise). Fixing this threshold not only reflects the inherent geometric properties of orchid leaves but also simplifies the algorithm structure, thereby enhancing computational efficiency and consistency.

Once the first three keypoints have been identified, the subsequent keypoints were determined by iteratively running the same search algorithm. For example, to determine $P_{4}^{1}$ , the circular search area is iteratively expanded with $P_{3}^{1}$ as the center. The unvisited points within the left semicircle of this area form a set $P_{4\left(1\right)}^{1},P_{4\left(2\right)}^{1},\ldots ,P_{4\left(N\right)}^{1}$ . The algorithm then calculates the angle set $S_{\theta }=\{{\hat{\theta }}_{i-1\left(k\right)}|{\hat{\theta }}_{i-1\left(k\right)}=∠P_{3}^{1}P_{2}^{1}P_{4\left(k\right)}^{1},k=1,2,\ldots ,N\}$ and ${\hat{\theta }}_{i-2}=∠P_{2}^{1}P_{1}^{1}P_{3}^{1}$ . It compares the differences between ${\hat{\theta }}_{i-2}$ and each ${\hat{\theta }}_{i-1\left(k\right)}$ . Similar to the selection of $P_{3}^{1}$ , if the smallest angle difference exceeds the threshold of 20°, the circular search area is further expanded. Otherwise, the point with the smallest angle difference was selected as $P_{4}^{1}$ , as follows


$$S2_{\theta }=\left\{\Delta \theta_{i-2\left(k\right)}|\Delta \theta_{i-2\left(k\right)}={\hat{\theta }}_{i-1\left(k\right)}-{\hat{\theta }}_{i-2},k=1,2,3,\ldots ,N\right\}$$

$$\text{min}S2_{\theta }\leq 20^{\circ }$$

$$i=argminS2_{\theta },P_{4\left(k\right)}^{1}\leftarrow P_{i+1\left(i\right)}^{1}$$

Then, the same method was iteratively applied to locate subsequent keypoints $P_{i}^{1}$ . The loop terminates under the following condition


$$\begin{array}{c} \left|\left|C_{pot}-P_{last}\right|\right|\leq r_{pot}+r_{last} \end{array}$$

where $P_{last}$ represents the last identified keypoints of the leaf, $r_{pot}$ is the radius of $C_{pot}$ , $r_{last}$ is the radius of the circular search area corresponding to $P_{last}$ , and $\left|\left|C_{pot}-P_{last}\right|\right|$ is the Euclidean distance between the centers of $C_{pot}$ and $P_{last}$ . When the circular search area of $P_{i}^{1}$ contains no unvisited points and intersects with $C_{pot}$ , $P_{i}^{1}$ is designated as $P_{last}$ , and the iteration stops. At this time, all keypoints for the single leaf have been determined.

The algorithm is then repeated on other leaves. The point $P_{1}^{2}$ , which has the greatest Euclidean distance from $C_{pot}$ , is identified as the first keypoints of the second leaf. The same algorithm was then applied to determine all keypoints of this leaf, continuing until the keypoints for all leaves were found.

In front view of orchids, due to the influence of gravity, all orchid leaves form a completely regular convex polyline (**Figure 3B**). Therefore, the trajectory pattern was fitted by minimizing curvature based on the consistent leaves trend. Let any three continuous keypoints along the leaf (from the root to the tip) be denoted as $P_{i-1}^{1}$ , $P_{i}^{1}$ , and $P_{i+1}^{1}$ . These points must satisfy the following condition


$$\{\begin{array}{l} P_{iy}^{1}>P_{\left(i-1\right)y}^{1}\\ P_{iy}^{1}>P_{\left(i+1\right)y}^{1} \end{array},\forall i\in \left[2,N_{j}-1\right]$$

where $N_{j}$ represents the number of keypoints on the j -th leaf. This ensures that any adjacent three keypoints form a convex sub-polyline, and the collection of these sub-polylines constitutes the fully convex skeleton of the leaf.

The keypoints identification process for the front view image of the orchid leaf begins by selecting the initial point $P_{1}^{1}$ as the farthest point from the leaf base. To determine the subsequent point $P_{2}^{1}$ , a circular search area centered on $P_{1}^{1}$ is iteratively expanded until more than two unvisited points were found. The point with the closest y -coordinate to $P_{1y}^{1}$ is then selected as $P_{2}^{1}$ . The next keypoint, $P_{3}^{1}$ , is identified by expanding the search area centered on $P_{2}^{1}$ . If more than 2 unvisited points were found within this area, the point that forms a convex curve with $P_{1}^{1}$ and $P_{2}^{1}$ and results in minimal curvature is chosen as $P_{\text{tem}1}^{1}$ .

The search then continues by expanding the area around $P_{\text{tem}1}^{1}$ . If there were at least two points in this area, they were evaluated. If a point $P_{\text{tem}2}^{1}$ forms a convex polyline with $P_{1}^{1}$ and $P_{2}^{1}$ and lies above $P_{\text{tem}1}^{1}$ , this indicates that the polyline $P_{1}^{1}P_{2}^{1}P_{\text{tem}2}^{1}$ has a lower curvature than $P_{1}^{1}P_{2}^{1}P_{\text{tem}1}^{1}$ . In this case, the following vector addition is performed as

$\vec{P_{2}^{1}P_{tem1}^{1}}+\vec{P_{tem1}^{1}P_{tem2}^{1}}=\vec{P_{2}^{1}P_{tem2}^{1}}$


$$P_{3}^{1}\leftarrow P_{\text{tem}2}^{1}$$

Thus, $P_{\text{tem}2}^{1}$ is set as the final $P_{3}^{1}$ , and $P_{\text{tem}1}^{1}$ is discarded. Conversely, if $P_{\text{tem}2}^{1}$ is located below $P_{\text{tem}1}^{1}$ , then $P_{\text{tem}1}^{1}$ is confirmed as $P_{3}^{1}$ , and $P_{\text{tem}2}^{1}$ becomes $P_{4}^{1}$ . This iterative traversal, combined with vector addition, allows for a more precise fitting of the leaf structure in front view.

All experiments—orchid (two industrial cameras) and maize (PHENOARCH platform)—were run with a single, immutable parameter set. Values were selected once on an orchid batch and were not tuned thereafter, thereby demonstrating cross-species generalizability. **Table 3** lists the constants, and no entry changes across datasets, resolutions or cameras.

**Table 3:** Global implementation parameters used for all images.

Parameter	Symbol description	Fixed value
Hue range (HSV)	$H_{min}-H_{max}$	35°-85°
Opening/closing kernel	B (square)	5×5
Erosion iterations	—	1
Contour area cut-off	$A_{\text{min}}$	500 px
Aspect-ratio filter	$AR_{\text{min}}$	>2
Sampling grid size	patches per side	40×40
Initial search radius	$r_{0}$	5 px
Angle-difference threshold	$\theta_{max}$	20°
Convexity constraint	three-point test	enforced

### 2.5 Evaluated indicators for skeletonization algorithms

**Figure 4:**
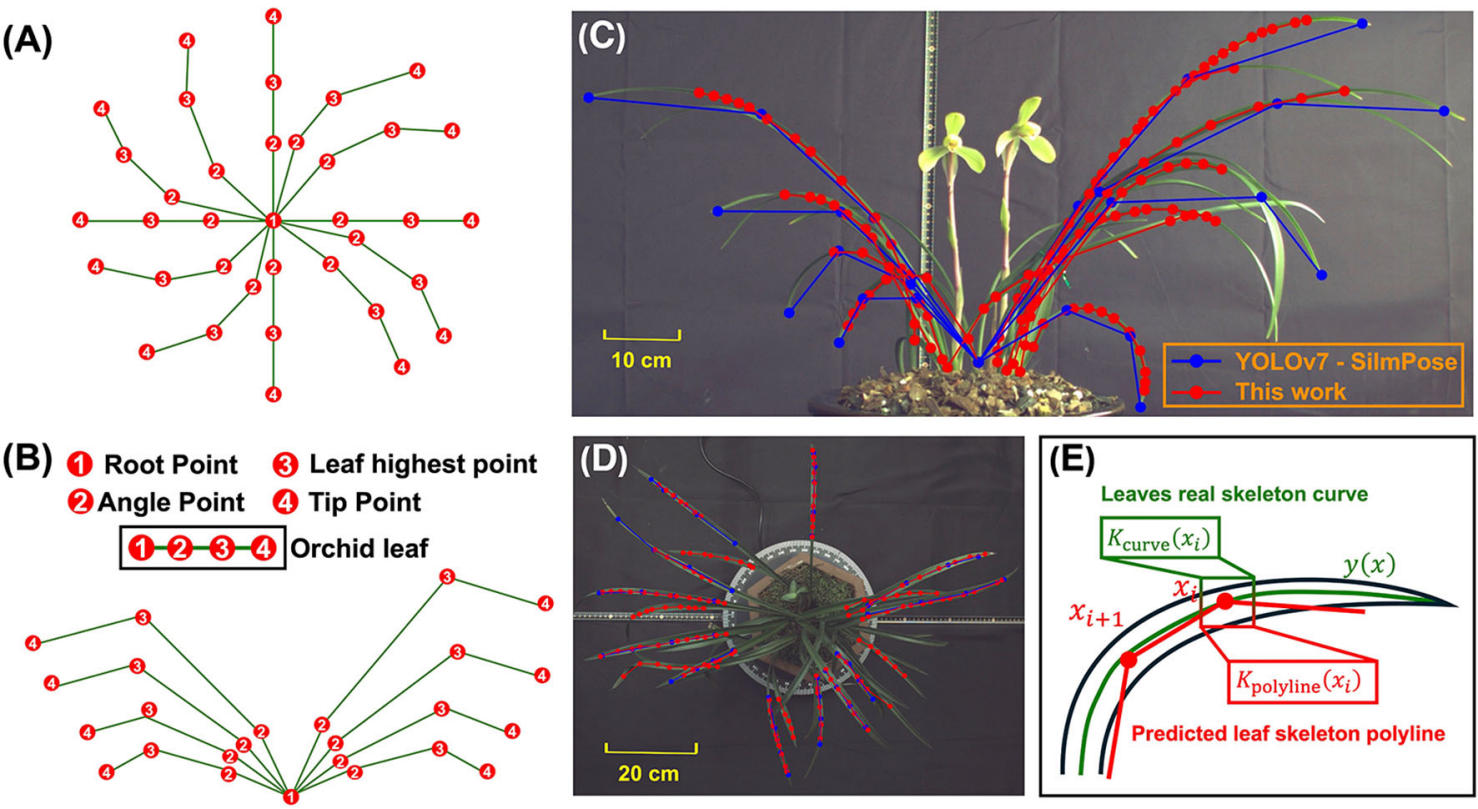
Predefined keypoints for YOLOv7-SlimPose in **(A)** vertical view and **(B)** front view. Identified keypoints and skeletons in **(C)** front view and **(D)** vertical view. **(E)** Curvature of real leaf skeleton curve and predicted leaf skeleton polyline at point *x_i_*.


The accuracy of the predicted skeletons was evaluated using curvature error (**Figure 4**). Let y(x) represent the true skeleton curve of the leaf, with curvature at point $x_{i}$ given by (Schamberger et al., 2023)

$$\kappa_{\text{curve}}\left(x_{i}\right)=\frac{\left|y^{\prime \prime }\left(x_{i}\right)\right|}{{\left(1+{\left(y'\left(x_{i}\right)\right)}^{2}\right)}^{\frac{3}{2}}}$$

and the curvature at point $x_{i}$ of the predicted leaf skeleton polyline is given by (Chuon et al., 2011)


$$\kappa_{\text{polyline}}\left(x_{i}\right)=\frac{\theta_{i-1}+\theta_{i+1}-2\theta_{i}}{d_{i-1,i}+d_{i,i+1}}$$

where $\theta_{i}$ represents the angle at the i -th vertex of the polyline, and $d_{i-1,i}$ is the Euclidean distance between the (i−1) -th and i -th points on the polyline. The curvature error between the predicted and true skeletons is defined as


$$\text{Curvature Error}=\frac{1}{N}\sum_{i=1}^{N}\left|\kappa_{\text{curve}}\left(x_{i}\right)-\kappa_{\text{polyline}}\left(x_{i}\right)\right|$$

A smaller curvature error indicates a higher shape conformity between predicted polyline skeleton and the true curve skeleton. The leaf recall rate was used to measure the proportion of leaves correctly identified and successfully skeletonized out of the total number of leaves, defined as


$$\text{Leaf recall rate}=\frac{\left|\left\{P_{1}^{j},j=1,2,\ldots ,N\right\}\right|}{\text{Ground Truth of leaves number}}\times 100\%$$

### 2.6 Leaves phenotypes

**Figure 5:**
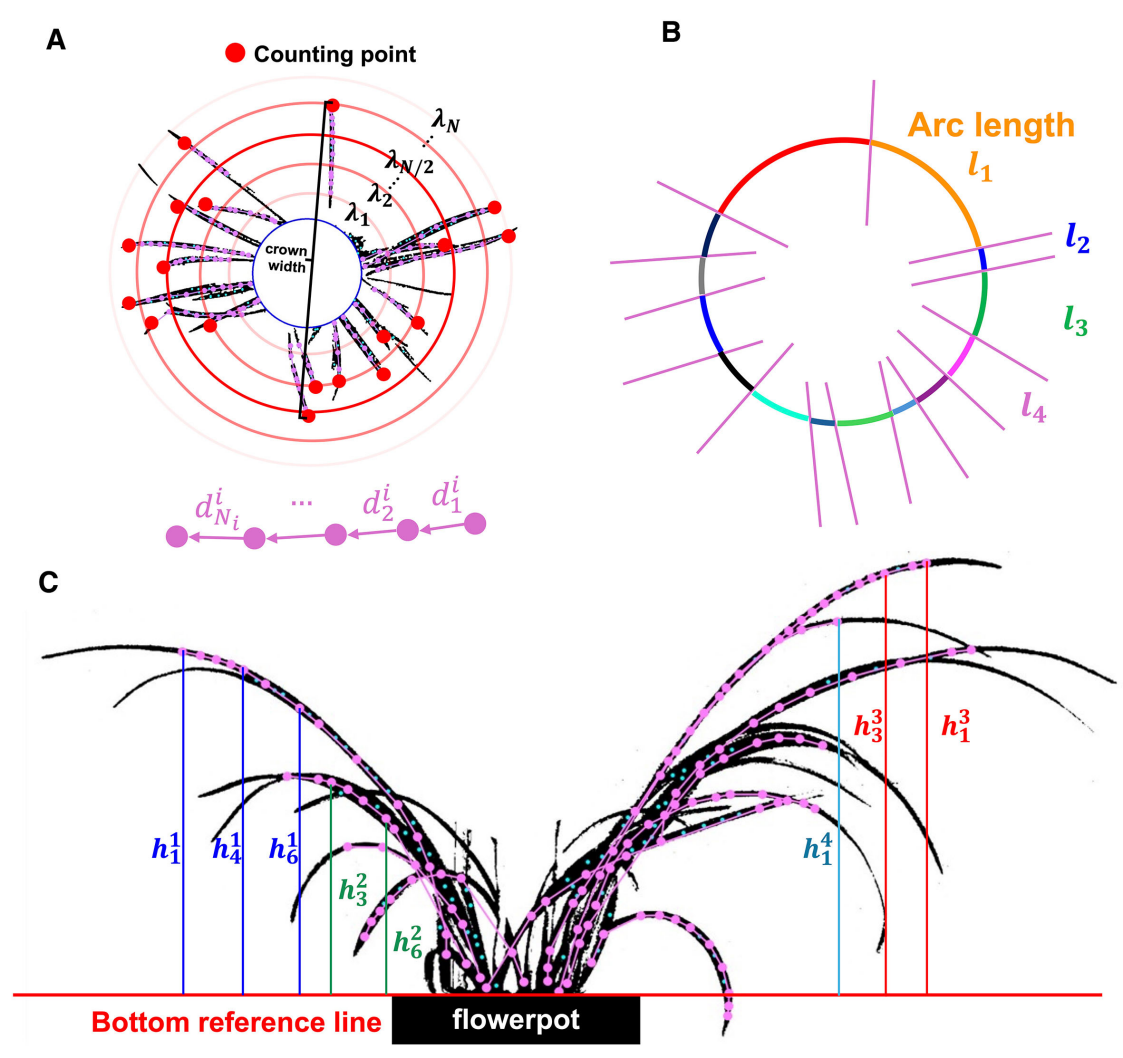
**(A)** The initial point $P_{i}$ located at the leave tip, and the crown width obtained from the two farthest initial points. **(B)** Arcs obtained by intersecting concentric circles with leaf skeleton. **(C)** The height of each keypoints relative to the bottom reference line in front view.


For vertical view, the number of algorithm’s outermost loop (which traverses each leaf) corresponds to the leaves’ number. As shown in **Figure 5A**, since the initial point $P_{1}^{j}$ for each leaf is closest to the leaf tip, the total number of $P_{1}^{j}$ represents the leaf count. The maximum distance between any two $P_{1}^{j}$ represents the crown width of the orchid (Ebrahimi et al., 2020), measured in pixels. Because pixel measurements cannot accurately reflect the true crown width, the relative crown width was used as a key feature for evaluating the orchid, formulated as

$$\text{Relative Crown Width}=\frac{\text{Crown Width}}{\text{Diameter of}\;C_{pot}}$$

Let $N_{j}$ represent the number of keypoints in the $j^{th}$ leaf. Since the skeleton formed by connecting these keypoints accurately represents the shape of the leaf, the total length of the skeleton corresponds to the length of the leaf. Leaf height consistency is the variance of each leaf’s mean vertical distance from keypoints to the pot’s top reference line in the front view, reflecting canopy regularity that growers use to judge form. Relative crown width is scale robust by construction. Leaf height consistency is expressed as


$$\text{Leaf Length Consistency}=\text{Var}\left(\sum_{k=1}^{N_{i}}d_{k}\right)$$

A concentric circle $C_{crown}$ is drawn with the crown width as its diameter, centered on the flowerpot (**Figures 5A, B**). Between $C_{crown}$ and $C_{pot}$ , N additional concentric circles $C_{j}$ (j=1,2,…,N) were generated with equal radial increments, where the radius of each $C_{j}$ is denoted as $R_{C_{j}}$ . The boundaries of each concentric circle may intersect the skeleton of the leaves at different points. Each intersection divides $C_{j}$ into several arcs, with $l_{i}^{j}$ representing the arc length of the i -th segment of the j -th concentric circle, corresponding to the distance between adjacent leaves under the condition of $R_{C_{j}}$ . Thus, $Var\left(l_{j}\right)$ represents the leaf distribution consistency at $R_{C_{j}}$ .

However, in vertical view, the leaves radiate outward from the center, causing the distance between adjacent leaves to vary under different $R_{C_{j}}$ conditions. Generally, $l_{i}^{j}$ is positively correlated with $R_{C_{j}}$ , and leaf distribution consistency at an intermediate $R_{C_{j}}$ better reflects the overall leaf uniformity of the orchid. Therefore, leaf distribution consistency for the orchid (the weighted average of $Var\left(l_{j}\right)$ ) quantified how uniformly leaves occupy space around the pot by partitioning the annulus between the pot circle and the crown circle into concentric rings. Leaf distribution consistency is defined as


$$\text{Leaf Distribution Consistency}=\sum_{j=1}^{N}\lambda_{j}Var\left(l_{j}\right)$$

where $\lambda_{j}$ is a Gaussian function satisfying $\sum_{j=1}^{N}\lambda_{j}=1$ and $argmax\left\{\lambda_{j}\right\}=\frac{N}{2}$ , expressed as


$$\lambda_{j}=\frac{\text{exp}\left(-\frac{{\left(j-\frac{N}{2}\right)}^{2}}{2\sigma^{2}}\right)}{\sum_{k=1}^{N}\exp\left(-\frac{{\left(k-\frac{N}{2}\right)}^{2}}{2\sigma^{2}}\right)}=\text{Softmax}\left(-\frac{{\left(j-\frac{N}{2}\right)}^{2}}{2\sigma^{2}}\right)$$

For front view, the top edge of the flowerpot’s rectangular boundary serves as the bottom reference line of leaves ( $l_{r}$ ). The distance from the i -th key point of leaf j to $l_{r}$ is denoted as $h_{i}^{j}$ , and the average distance of all keypoints in leaf j to $l_{r}$ is represented by ${\hat{h}}_{j}=\frac{1}{M_{j}}\sum_{k=1}^{M_{j}}h_{k}$ . The variance of ${\hat{h}}_{j}$ across all leaves reflects the variation in the average height of each leaf within the orchid, which is defined as leaf height consistency (**Figure 5C**), indicating whether the architecture is harmonized rather than a mix of very long and very short leaves. Leaf height consistency is formulated as


$$\text{Leaf Height Consistency}=Var\left(\frac{1}{M_{j}}\sum_{k=1}^{M_{j}}h_{i}\right)$$

where $M_{j}$ represents the number of keypoints on leaf j in front view.

## 3 Results and discussion

### 3.1 Keypoints connection results and phenotypic extraction

**Figure 6:**
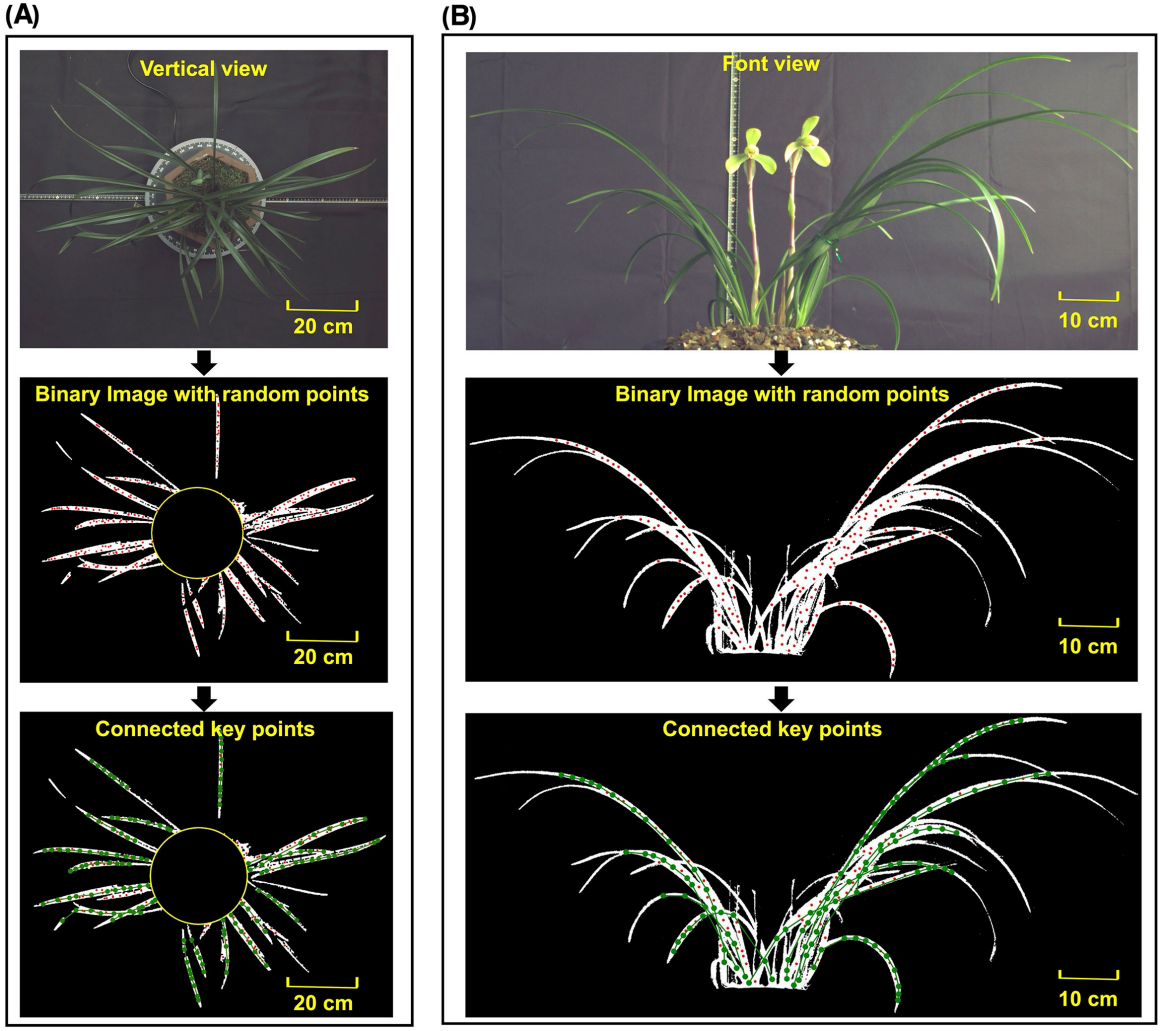
Original image, binary image with random points and binary image with connected keypoints in **(A)** vertical view and **(B)** front view.

The keypoints connection method was applied to both front view and vertical view images of the orchid. The keypoints connection result was shown in **Figures 6A, B**, respectively It can be seen that the leaves were accurately extracted without any background noise. Due to the application of morphological erosion to reduce the leaf boundary regions, random points were uniformly generated in areas close to the central skeleton. Since the leaf tip width is narrow, the diameter of the random points exceeded the eroded width of leaf tip, resulting in incomplete point generation at the outermost tip. After executing the keypoints algorithm, nearly all leaf skeletons were accurately extracted under complex conditions involving dense leaves, leaf intersection and leaf occlusion. Motivated by this observation, a quantitative analysis was conducted to explicitly characterize when tip detection fails and how this affects downstream phenotypes.

To make failure modes explicit, a leaf-level criterion was adopted whereby a tip is counted as missed if the outermost 20% sector of that leaf contains no sampled points. Using this definition and expressing the random-point marker diameter as a percentage of the shortest contour length in $S_{cont}$ , the tip-miss frequency over the entire orchid dataset (front and top views combined) was 19.24% at the default setting of 1.5%. An ablation varying the marker diameter showed a monotonic decrease in misses—from 32.34% (6%) to 0% (0.1%)—indicating that erosion-induced narrowing at very thin tips is the primary cause and can be mitigated by smaller markers. Phenotype level effects were limited at the default: leaf count was essentially unchanged. Relative crown width exhibited a mild negative bias (−2.4%). Leaf-length consistency showed a small positive bias (+2.6%). Leaf distribution consistency changed negligibly due to Gaussian mid-radius weighting. And in front views, leaf-height consistency showed a minor negative bias (−1.1%). These results indicate that, under default settings, tip misses occur in a fraction of leaves but have minor impact on the five target phenotypes. If complete tip recovery is required, reducing the marker diameter (≤0.3%) eliminates the effect without altering the rest of the pipeline.

YOLOv7-SlimPose (Gao et al., 2024) was utilized based on the same dataset. For each leaf, four keypoints were predefined and annotated (**Figures 4A, B**): (1) Root point, which is the point where the base of the plant connects to the pot; (2) Angle point, located at the midpoint between the Root point and the Tip point; (3) Angle point 2, positioned at three-quarters of the distance from the Root point to the Tip point; and (4) Tip point, representing the apex of the leaf. Keypoints point (1) through (4) were sequentially connected to form the leaf skeleton. 10 leaves and 8 leaves were annotated in vertical view images and font view images respectively. The annotated leaf skeletons in each image were evenly distributed. A total of 561 images were selected for the training set, 126 images for the validation set, and the remaining 15 images were used as the test set. The ratio of vertical view to front view images was maintained at 1:1 across the training, validation, and test sets. Due to the distinct distribution of leaves in the vertical and front view, the images from these two perspectives were separated and used to train two models: one for vertical view and another for front view (Total training time is 7.3 hours). After training, the test set images were input into YOLOv7-SlimPose, which output the leaf keypoints and skeletons for front view (blue output in **Figure 4C**) and vertical view (blue output in **Figure 4D**).

**Figure 7:**
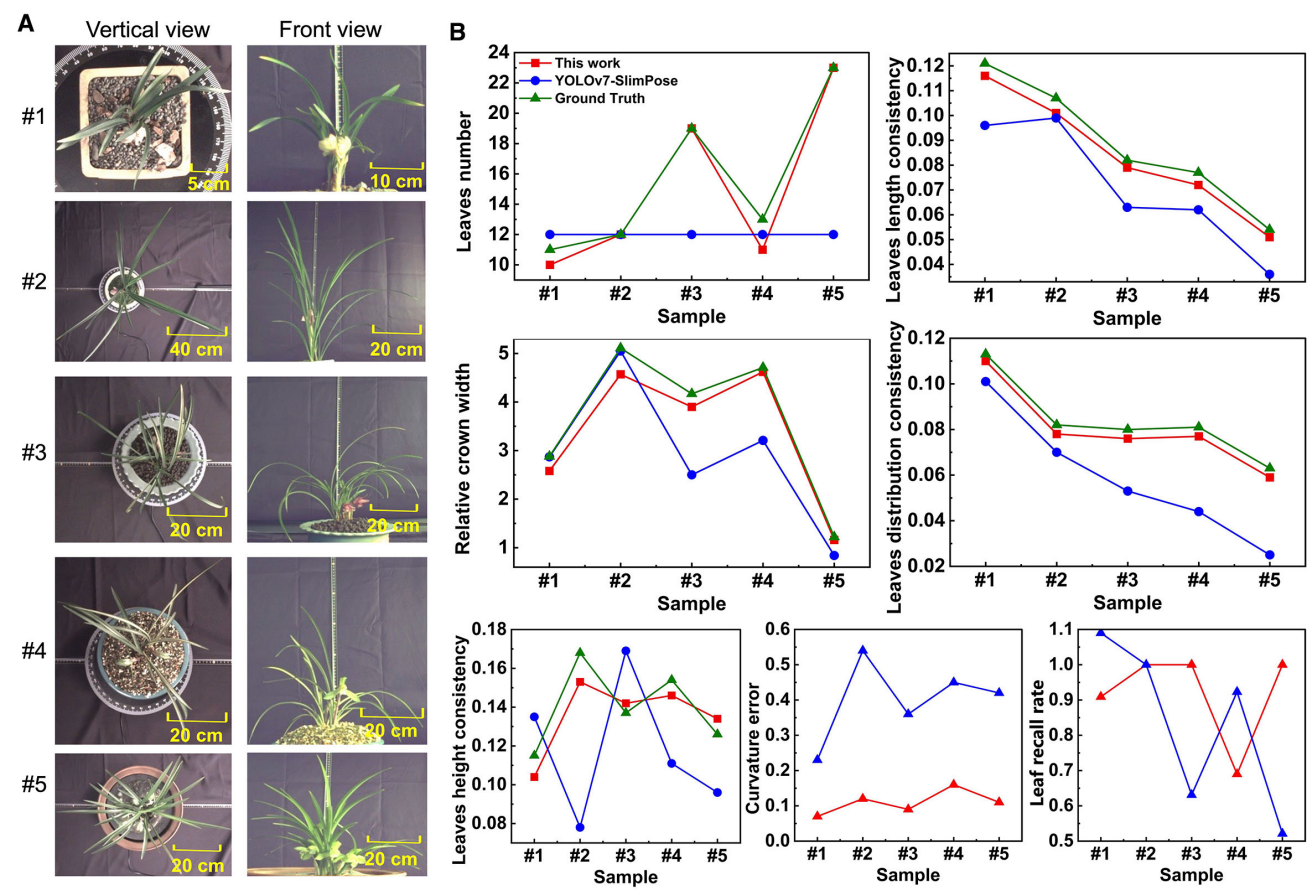
**(A)** Vertical view and front view in test set (5 samples as example). **(B)** 5 phenotypes, curvature error and leaf recall rate obtained by YOLOv7-SlimPose and this work.

The test set images (**Figure 7A**) were processed by both algorithms. Based on the identified keypoints and the leaf skeleton, 5 key features were further extracted, and the curvature error and leaf recall rate were calculated for each, as shown in (**Figure 7B**).

YOLOv7-SlimPose has a fixed output layer structure and relies on an anchor-based object detection mechanism, which restricts the size and position of the output targets within a predefined range (Liu et al., 2022). The model generates a fixed number of keypoints for each anchor position during forward propagation. This design limits the model to outputting only a predetermined number of keypoints and predefined skeleton connections (He et al., 2022; Tan et al., 2024). Consequently, skeletons’ number and keypoints detected by YOLOv7-SlimPose model are entirely dependent on the annotations in the training data, which often require expert knowledge in botany, especially when dealing with complex plant structures.

For front view, orchid leaf root points were distributed across various positions within the pot. However, due to annotation constraints, YOLOv7-SlimPose identifies all root points as being concentrated in a single location, failing to represent the true morphology of leaves. Orchids exhibit varying numbers of leaves, leaf lengths, and leaf distributions, but YOLOv7-SlimPose lacks the flexibility to dynamically adjust the output structure based on the input, making it inadequate for handling uncertain keypoints numbers and connection patterns. Therefore, deep learning models like YOLOv7-SlimPose are more suitable for applications involving fixed keypoints numbers and skeleton structures, such as human or animal pose estimation, rather than for complex plants like orchids, which have dense and variable leaf pattern.

Based on the skeletons generated by YOLOv7-SlimPose, five orchid phenotypes were extracted from the test set, with the results shown in **Figure 7**. For the vertical view, as the annotated number of leaves was fixed at 12, the model consistently detected 12 leaf skeletons (with the leaf count remaining constant at 12) and 37 keypoints across all images. In contrast, the method proposed in this work accurately detected varying numbers of leaves, achieving a leaf recall rate of 1 in three out of five samples. In the first two samples, where the leaves were relatively sparse, YOLOv7-SlimPose was able to detect most of the leaf tips, resulting in a relative crown width close to the ground truth. However, as the number of leaves increased in the remaining samples, the accuracy of the crown width predictions by YOLOv7-SlimPose dropped significantly compared to this work. The fixed number of keypoints output by YOLOv7-SlimPose resulted in poor fitting of the true leaf skeleton curve, limiting the model’s ability to accurately reflect leaf consistency. The average curvature error and leaf recall on test set were only 59% and 63%, respectively. In contrast, this work successfully detected enough leaves and keypoints, and accurately fitted the real leaf skeleton, resulting in more precise extraction of leaf length consistency, distribution consistency, and height consistency. Without manual labeling and long-term training, the average curvature error and blade recall of the test set reached 0.12 and 92%, respectively.

**Figure 8:**
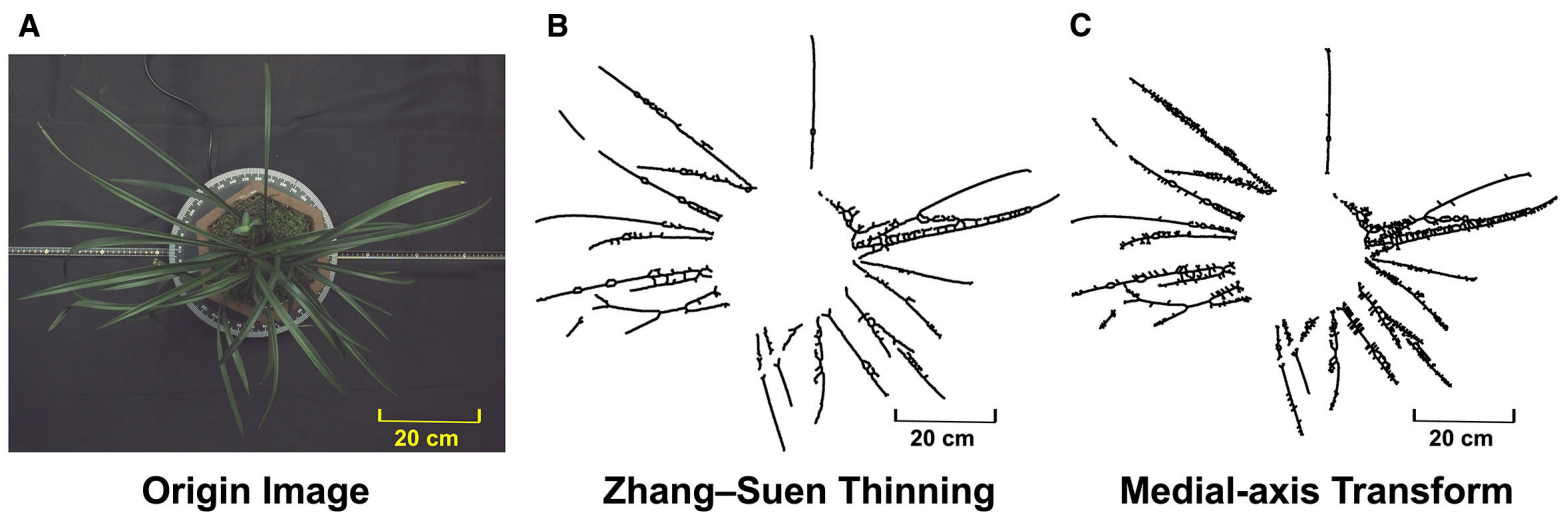
**(A)** Original image; **(B)** skeletonization result of Zhang–Suen thinning; **(C)** skeletonization result of MAT.

To demonstrate how the proposed spontaneous key-points connection differs from traditional non-learning approaches, the same binary masks were processed with the medial-axis transform (MAT) and with Zhang–Suen thinning (**Figure 8**). Both classical algorithms inherit every imperfection present in the thresholded image: darker leaf segments translate into complete breaks, while small contour irregularities give rise to dense clusters of spurs and lateral branches. The resulting skeletons are fragmented and highly branched, preventing the continuous center-line that downstream phenotype measurements require. By contrast, the proposed strategy links interior sample points under geometric constraints that actively suppress burr formation and bridge minor gaps, yielding a single, smooth curve for each leaf and enabling reliable extraction of length, curvature and spatial-distribution traits. The qualitative comparison therefore reinforces the practical advantage and uniqueness of the present method for complex leafy plants.

### 3.2 Computational efficiency or practicality

Experiments were conducted on a CPU-only workstation equipped with 32 GB DDR4–2666 system memory. For images of 4024 × 3036 and 3072 × 2048 pixels, the algorithm required on average 3.556 s and 2.247 s per image, respectively, with peak resident memory of approximately 190.50 MB and 150.37 MB. No GPU acceleration was used.

Compared with the classical methods that have been publicly disclosed, iterative refinement in the i5-4670K and 4 GB memory virtual machine environment, most algorithms are completed within the range of 0.58-0.96 s for a single image, while Stentiford and an improved method take about 1.21-1.43 s (Gramblicka and Vasky, 2016). The total runtime given in the example script, represented by the central axis transformation, is about 1.34 s/image. The total runtime of the activity outline in similar official examples is about 2.03 seconds per image. The Hessian Frangi ridge filter shows an average of 1.187 s/graph in the i7 3.0 GHz, 64 GB, RTX 2060 environment, and hardware configuration is provided to ensure comparability (Hachaj and Piekarczyk, 2023).

Although the single frame time is slightly higher than several classic skeleton pipelines, this method does not require labeling and training, and can adaptively generate a variable number of keypoints and skeletons after one preprocessing. It directly supports phenotype calculations such as leaf number, crown width, length consistency, distribution consistency, and height consistency, and has been validated for stable leaf recall rate and cross species generalization in orchid and maize morphologies. The practicality of this type of “training species independent” is supported by the cost of running a CPU in seconds and a hundred megabits of memory, avoiding the time and economic expenses of retraining and manual annotation. Regarding scalability, when the number of pixels increases from 6.29 million to 12.22 million (1.94 ×), the average time takes from 2.247 s to 3.556 s (1.58 ×), showing a nearly linear or even better scaling. The algorithm mainly relies on local search and angle/convexity constraints on the mask, and the computational complexity mainly increases linearly with the number of pixels and candidate points, so it will only be faster at lower resolutions. 4024 × 3036 has significantly exceeded the imaging requirements of most production processes, and high-resolution is chosen to cover the most demanding resolution scenes. In terms of density, the high leaf density, occlusion, and overlap of orchids have constituted strict stress tests, while the full growth period samples of corn gradually increase leaf density, all of which can maintain stable skeleton extraction and high leaf recall rate, indicating that the scalability in leaf quantity and density has practical significance.

### 3.3 Generalization ability verification

**Figure 9:**
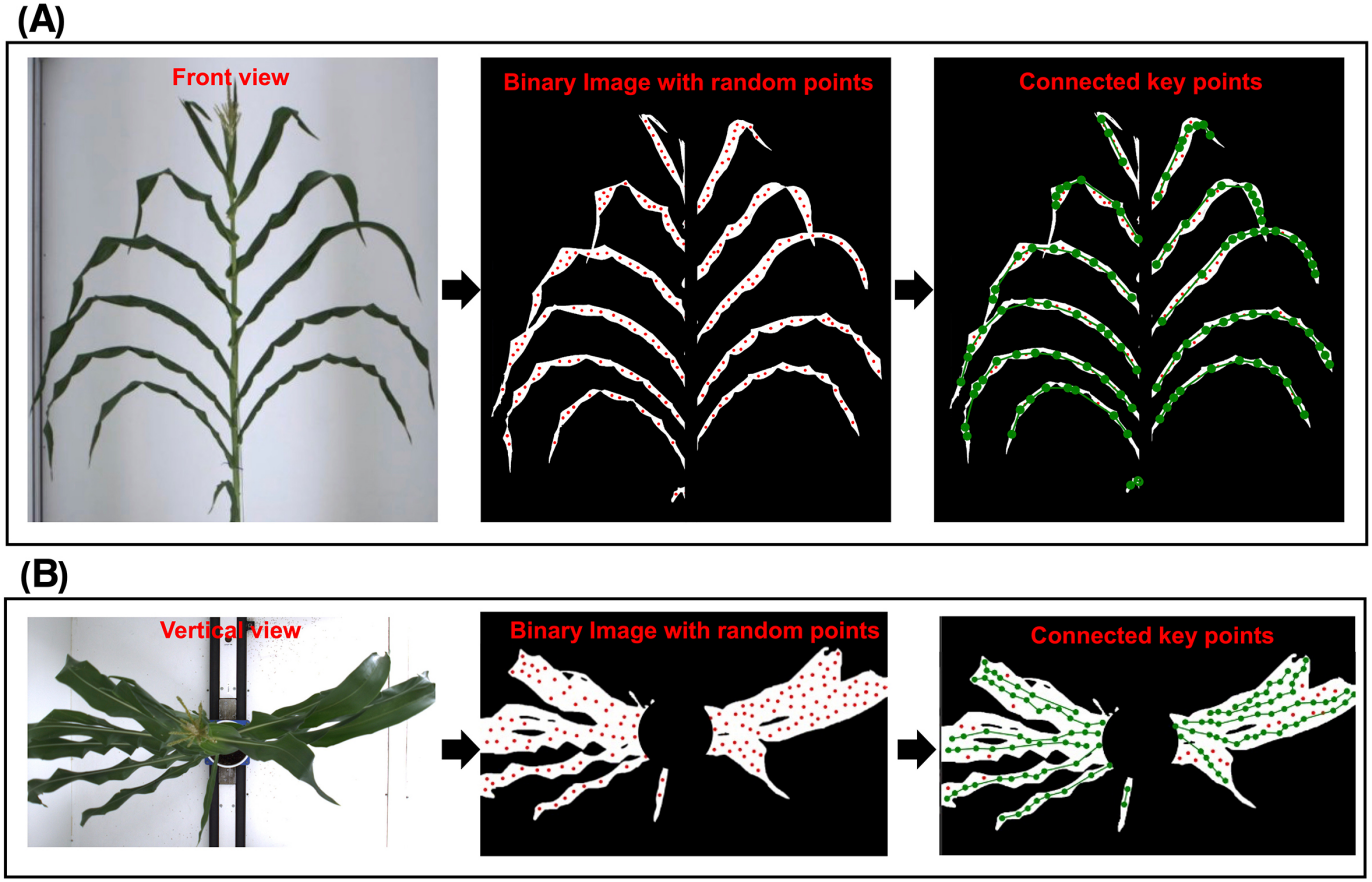
Original image, binary image with random points and binary image with connected keypoints of a single maize in **(A)** vertical view and **(B)** front view.

**Figure 10:**
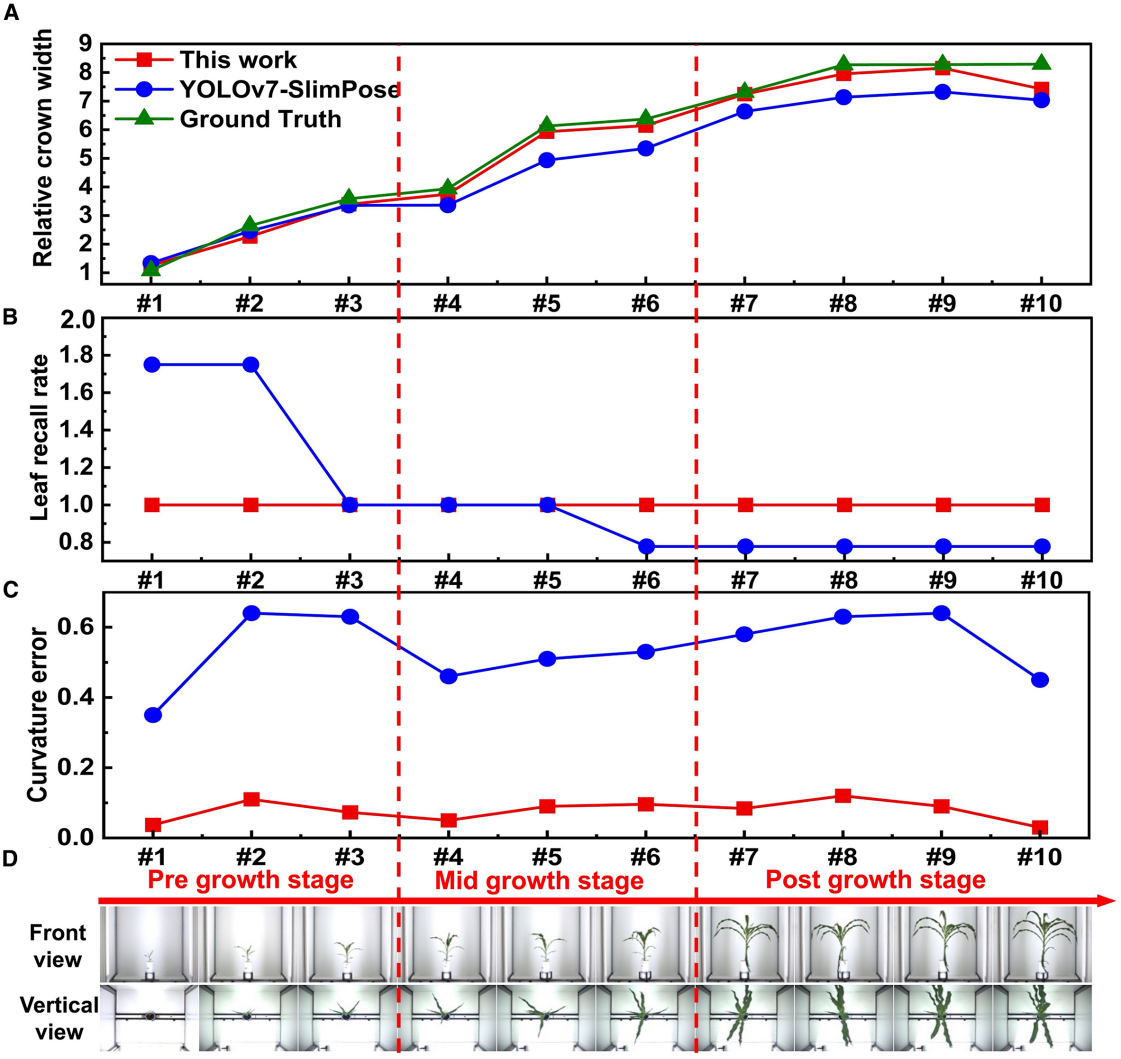
**(A)** Relative crown width, **(B)** curvature error and **(C)** leaf recall rate of a single maize plant throughout entire growth stage obtained by YOLOv7-SlimPose and this work. **(D)** Vertical view and front view images of a single maize plant throughout entire growth stage.

To verify the generalization ability of the proposed algorithm, it was applied to maize. Similar to orchid leaves, maize leaves are soft, numerous, densely packed, and exhibit significant overlap. Using the publicly available single-plant maize dataset (Dataset URL: https://datasetninja.com/maize-whole-plant-image-dataset), which captured images of a single maize plant over 113 days—with 1 vertical view and 12 front view images taken each day—the spontaneous keypoints connection algorithm was applied to both a front view (**Figure 9**) and a vertical view image (**Figure 9**) from 10 different days, spanning pre-, mid-, and post-growth stages (**Figure 10**). Due to the relatively wide leaves of maize, random points could still be generated at the eroded leaf tips, and in some cases, a single maize leaf may be mistakenly identified as two separate leaves in the vertical view.

It is important to note that the extraction of maize leaf count is based on the front view images, while the vertical view is primarily used to extract features such as crown width, leaf distribution consistency, and overall plant structure. Therefore, any misidentification of leaf number in the vertical view does not affect the final feature extraction or the evaluation of the algorithm’s generalization ability on maize leaves.

Samples #4 and #5 were annotated for YOLOv7-SlimPose training, with 6 skeletons annotated per front view image and 4 skeletons per vertical view image. The relative crown width was extracted from 10 samples (**Figure 10**). During the early growth stage, when maize leaves are smaller and fewer in number, YOLOv7-SlimPose predicted more skeletons than the actual leaf count, allowing it to accurately estimate the crown width in this stage. However, as the plant continued to grow, both the size and number of leaves increased. The number of leaves exceeded the prediction range of YOLOv7-SlimPose in mid-to-late growth stages, resulting in greater prediction error for the relative crown width.

In contrast, the proposed method accurately predicted the relative crown width across all growth stages (**Figure 10**) and consistently detected all leaves from the front view, maintaining a leaf recall rate of 100% throughout (**Figure 10**). Additionally, compared to YOLOv7-SlimPose, the proposed method maintained a lower curvature error (**Figure 10**) for front view images across all growth stages. These results confirm that the proposed method accurately extracts all keypoints and skeletons of maize leaves, demonstrating its effectiveness in keypoint detection, skeleton recognition, and feature extraction in crops with complex morphologies.

In summary, this work identifies keypoints and skeletons based solely on the connection of randomly generated points in the leaf region. This approach eliminates the need for predefined keypoints and training based on plant structure, making it adaptable for a broader range of plant phenotypic extraction tasks.

## 4 Conclusion

A spontaneous keypoints connection skeletonization algorithm based on random points was firstly developed for leafy plants phenotypic extraction. HSV color thresholding separated orchid leaves into binarized images. Morphological opening and closing operations, along with area and leaf shape filters, effectively removed noise. After leaf boundary erosion, random points were sampled near the central skeleton within each generated patch. The skeletonization approach was applied to plants with random leaf morphology and regular leaf morphology. For random leaf morphology, the point furthest from the center was identified as the first keypoint. A circular search was then iteratively expanded from this keypoint to find the next point. The subsequent keypoints search direction was determined based on the relative position of these two keypoints, thereby reducing the spatial complexity by half. Remaining keypoints were identified using angle difference thresholds. For regular leaf morphology, the algorithm ensured the accuracy of leaf skeleton fitting by enforcing that any three consecutive points formed a convex polyline while minimizing curvature. By iteratively applying this approach, nearly all leaves’ skeletons were detected, achieving an average curvature error of 0.12 and a leaf recall rate of 92%. In addition, a qualitative comparison with classical non-learning baselines showed that both inherit binarization discontinuities and produce fragmented, highly branched center-lines, whereas the proposed strategy yields smooth and continuous skeletons that enable downstream phenotypic measurements. Finally, five phenotypes of orchids were accurately extracted based on the identified skeletons. Moreover, this algorithm effectively predicted maize’s relative crown width throughout all growth stages and consistently detected 100% of the leaves, highlighting its generalization capability on wide leaf plants. Compared to existing literature reports, this approach accurately skeletonized leafy plants with random and regular morphological leaves, eliminating manual keypoints design and training based on plants’ structure. It can spontaneously detect keypoints and extract skeletons according to plant morphology, offering an effective solution for accurate phenotypic extraction of more plants with complex morphologies.

## Data Availability

The original contributions presented in the study are included in the article/supplementary material. Further inquiries can be directed to the corresponding author.
